# A Surprising Cause of Contained Aortic Rupture: Perforation from a Lumbar Osteophyte

**DOI:** 10.1055/s-0040-1715468

**Published:** 2021-03-24

**Authors:** Nikolaos Kontopodis, Christos V. Ioannou

**Affiliations:** 1 Vascular Surgery Unit, Department of Cardiothoracic and Vascular Surgery, University Hospital of Heraklion, Heraklion, Greece

**Keywords:** contained aortic rupture, pseudoaneurysm, osteophyte

## Abstract

We describe a patient with contained aortic rupture due to perforation from a protruding lumbar osteophyte, who was treated by open surgery. This case underlines that less common aortic pathologies are possible, which require a high suspicion index to be diagnosed.

Degenerative abdominal aortic aneurysm (AAA) is the most common type of AAA. Infection, dissection, and trauma are much less frequent causes of AAA development. Traumatic AAAs are in fact contained ruptures due to aortic perforation resulting in local containment of hemorrhage rather than uncontrolled bleeding. We describe a patient who presented with a traumatic aortic pseudoaneurysm due to perforation from a lumbar osteophyte.

A 75-year-old male patient was referred because of an incidentally discovered 5-cm AAA, which was identified on an ultrasound done for other reasons. The patient subsequently underwent computed tomography (CT) imaging, where a 5.7-cm infrarenal AAA with irregular wall boundaries was seen. A very distinctive appearance compared with the typical fusiform AAAs was found ( Fig. 1 ).

**Fig. 1:**
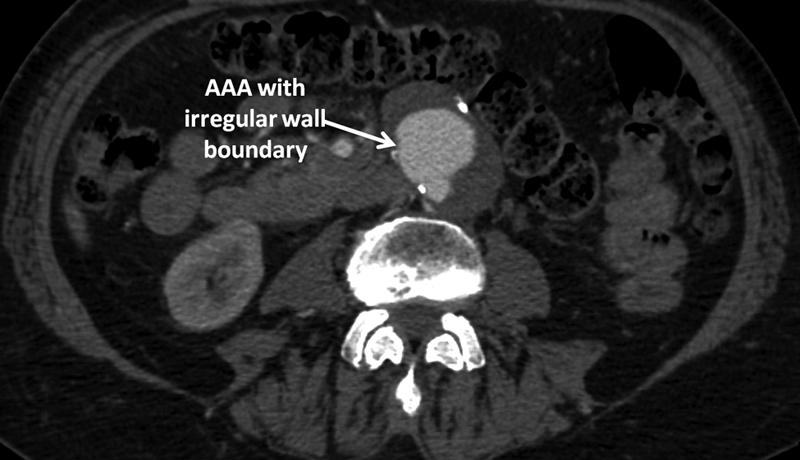
Axial computed tomography image. AAA, abdominal aortic aneurysm.

The patient was admitted and operated via open surgery. Endovascular options were not considered for this case because we were concerned that the irregular appearance of the lesion could be related to an infectious process. Intraoperatively, the unusual appearance of the lesion was obvious ( Fig. 2 ). After sac incision, a posterior aortic wall perforation became apparent from a lumbar osteophyte protruding into the lumen ( Fig. 3 ). The arterial lesion was resected, while the osteophyte was trimmed to avoid compression of the new interposition graft (expanded polytetrafluoroethylene, 16 mm) that was implanted. The postoperative course was uncomplicated.

**Fig. 2:**
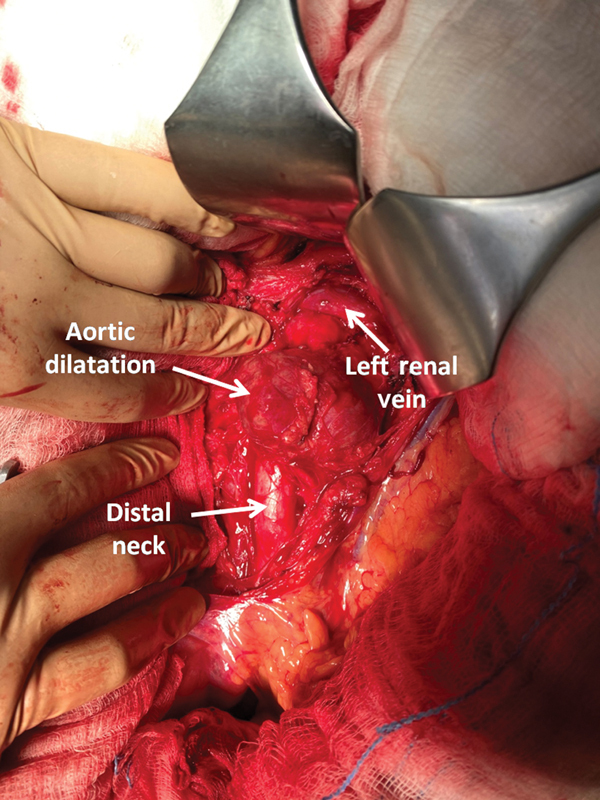
Intraoperative appearance of the lesion.

**Fig. 3:**
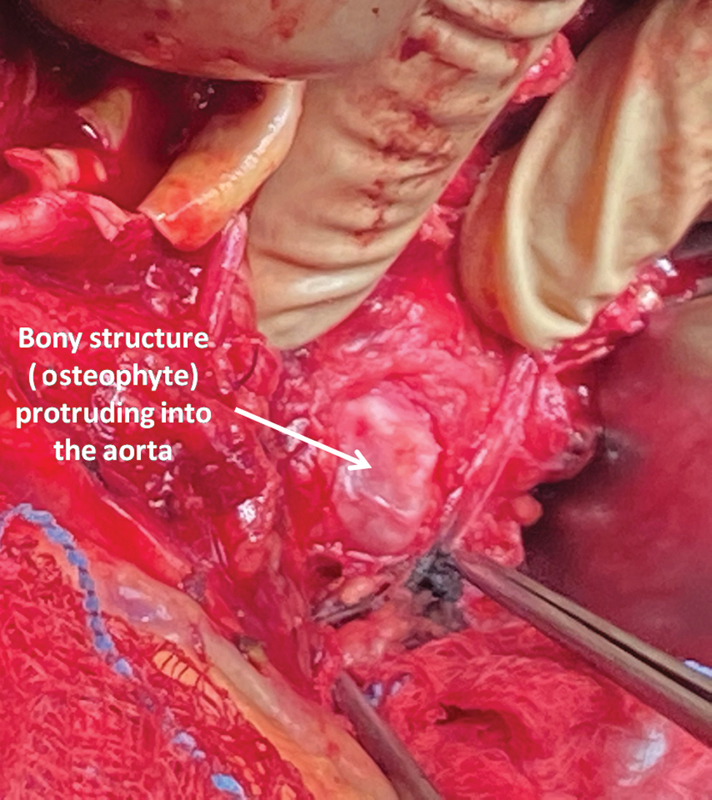
Intraoperative photo after incising the infrarenal aorta, showing the protrusion of the osteophyte into the posterior aortic wall.

Retrospective examination of the CT angiography identified the bony structure responsible for aortic perforation (L2 vertebra), which had not been apparent initially ( Figs. 4 and 5 ). The patient reported chronic back pain and an incident of pain exaggeration 20 days ago which was treated with anti-inflammatory medication.

**Fig. 4:**
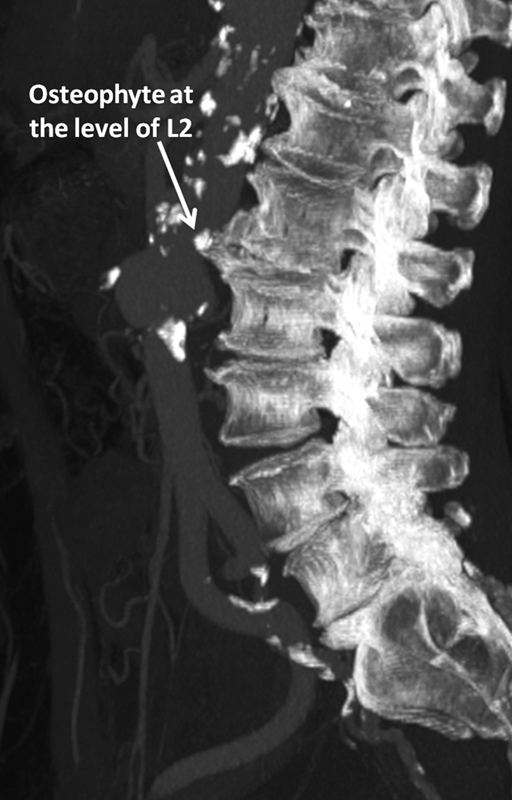
Sagittal image after multiplanar reconstruction.

**Fig. 5:**
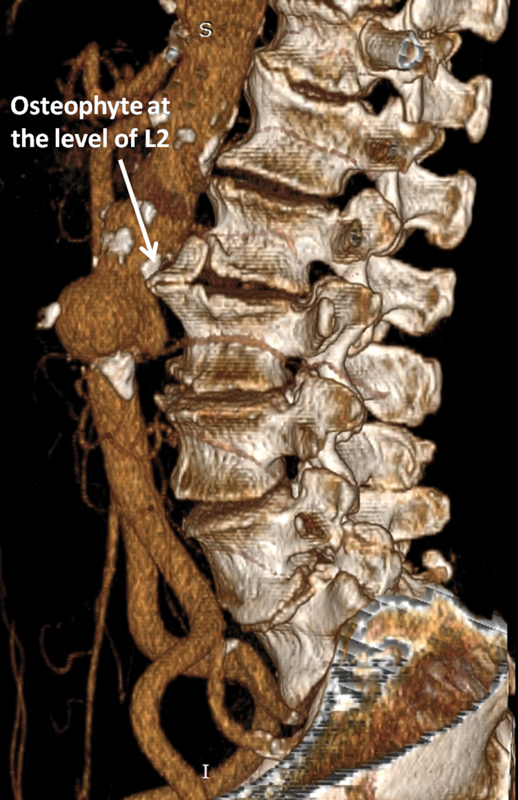
Three-dimensional reconstruction showing the long osteophyte (L2 vertebra) in direct contact with the posterior aortic wall at the level of contained rupture.

To the best of our knowledge, this is the first reported case of unprovoked aortic perforation from a lumbar osteophyte, while there have been a few reports of a similar lesion after blunt trauma. 1 2
